# Beacon-Based Remote Measurement of Social Behavior in ASD Clinical Trials: A Technical Feasibility Assessment

**DOI:** 10.3390/s21144664

**Published:** 2021-07-07

**Authors:** Lito Kriara, Joerg Hipp, Christopher Chatham, David Nobbs, David Slater, Florian Lipsmeier, Michael Lindemann

**Affiliations:** 1 Roche Pharma Research and Early Development (pRED), Digital Biomarkers, Roche Innovation Center Basel, 4070 Basel, Switzerland; david.nobbs.dn1@roche.com (D.N.); david.slater@roche.com (D.S.); florian.lipsmeier@roche.com (F.L.); michael.lindemann@roche.com (M.L.); 2 Roche pRED, Neuroscience and Rare Diseases, Roche Innovation Center Basel, 4070 Basel, Switzerland; joerg.hipp@roche.com (J.H.); christopher.chatham@roche.com (C.C.)

**Keywords:** autism spectrum disorder, Bluetooth low energy, clinical trials, digital biomarker, digital health technology tool, remote patient monitoring, sensor-based measure, sociability

## Abstract

In this work, we propose a Bluetooth low energy (BLE) beacon-based algorithm to enable remote measurement of the social behavior of the participants of an observational Autism Spectrum Disorder (ASD) clinical trial (NCT03611075). We have developed a mobile application for a smartphone and a smartwatch to collect beacon signals from BLE beacon sensors as well as to store information about the participants’ household rooms. Our goal is to collect beacon information about the time the participants spent in different rooms of their household to infer sociability information. We applied the same technology and setup in an internal experiment with healthy volunteers to evaluate the accuracy of the proposed algorithm in 10 different home setups, and we observed an average accuracy of 97.2%. Moreover, we show that it is feasible for the clinical study participants/caregivers to set up the BLE beacon sensors in their homes without any technical help, with 96% of them setting up the technology on the first day of data collection. Next, we present results from one-week location data from study participants collected through the proposed technology. Finally, we provide a list of good practice guidelines for optimally applying beacon technology for indoor location monitoring. The proposed algorithm enables us to estimate time spent in different rooms of a household that can pave the development of objective sociability features and eventually support decisions regarding drug efficacy in ASD.

## 1. Introduction

Autism spectrum disorder (ASD) is a highly prevalent neurodevelopmental disorder estimated to affect 1 in 88 children in the United States [1]. Individuals with ASD are highly heterogeneous in the type and severity of their core and associated symptoms, making the development of outcome measures challenging. The core symptoms of autism are social communication deficits and restricted and repetitive behaviors [2].

Current standards in assessing sociability in the context of clinical studies of ASD are based on clinician, caregiver and patient reported outcomes collected during clinical visits, such as the Vineland Adaptive Behavior Scales (VABS-III), Social Responsiveness Scale (SRS), Repetitive Behaviors Scale-Revised (RBS-R) [3]. Common problems with these tools include recall bias, poor inter/intra rater reliability, poor sensitivity to change, patient burden and lack of ecological validity [3]. These measurement problems may hinder the development of new treatments. In particular, subjective reporting may be associated with placebo responses that could mask treatment-related improvement [4].

Digital health technology tools may allow one to characterize different aspects of sociability during a participant’s daily life with objective, high-frequency measurements that may be more reliable, lower burden and more sensitive to change than the participant’s clinical measures and patient reported outcomes collected in the few clinical visits throughout a clinical trial. For example, in the outdoor environment, GPS can be used to identify time spent in “social places” [5] and Bluetooth can be used to infer the physical proximity of people [6]. In the home environment, sociability may be characterized using Wi-Fi [7], cameras using computer vision [8], Bluetooth [9,10,11], or combinations of multiple sensors such as RFID and cameras [12]. Each technology comes with different challenges. Firstly, cameras require an approach that preserves patient privacy which increases operational complexity, whereas RFID/Wi-Fi-based systems usually record the presence of a mobile sensor in relation to the different static RFID or Wi-Fi devices, increasing the system complexity of retrieving and combining the information from all devices for analysis. Bluetooth low energy (BLE) beacons, known mainly for applications in marketing [9], entertainment [10], airports [11], museums [13] or hospitals [14], do not have any of these constraints. BLE beacons transmit signals that can be recorded by one mobile sensor (e.g., a smartwatch), avoiding the need to clock synchronize all devices in the system, combine data recorded at multiple sources and do not transmit any personally identifiable data. This makes BLE beacons the easiest to deploy by non-technical people and with the ability to provide room level location estimation with the appropriate algorithm.

Other than the technology, another challenge we face is that a home setting is a much more dynamic environment compared to a shopping/entertainment center [9,10,11,12,13], given the smaller spaces, furniture placement, and variety of building materials used. Different homes have different layouts (e.g., very small rooms or open spaces), different materials used for the walls and furniture that have a different impact on the BLE beacon signal. Thin wooden walls introduce very low signal attenuation relative to a concrete wall, making the signal strength of neighboring rooms very similar. It is therefore difficult to differentiate the dominant BLE signal and identify the current room the person is in. Finally, metallic furniture/materials can increase signal reflection, introducing error again in the room estimation [7]. All these factors need to be accounted for in selecting the optimal technology and algorithm that can best support our application of identifying the room the clinical trial participant is in during the course of the study.

In this manuscript, we describe a solution using BLE beacons and evaluate the feasibility of performing in-home room-tracking (i.e., identify the room the person is in) in the context of a clinical trial. The proposed solution measures the proportion of time spent in rooms labeled (by the participant’s caregiver depending on the use of different rooms in the household) as social, not social or sometimes social. These measurements are taken as indicators of the sociability of the participant.

Firstly, we present our methodology with the technology we chose to use for this use case of indoor location estimation, the two studies (internal study with healthy volunteers, and a clinical trial with both typically developing and people with ASD) we ran to collect data, and we also describe the algorithmic steps we took in order to transform BLE signal to room location estimations for each participant across time for anyone to recreate.

Next, we present results showing that our proposed system and algorithm have an average accuracy of over 90% in predicting the correct room location when compared to ground truth information collected during our internal study. Moreover, we discuss signal collection challenges, scenarios that can decrease the accuracy of such location estimation systems, and we quantify the impact of these different scenarios. Moving to the clinical trial data information, we show that indeed participants were able to set up the technology on their own in their households without any technical assistance, but only with simple instructions from the clinician (with 96% of them setting up the BLE sensors during the first day of data collection). We also present the results of our proposed algorithm on two participants with ASD from the clinical trial. We show that there are patterns of sociability behavior over a week, demonstrating that in-home room-tracking can provide valuable insights into the everyday life of people with ASD.

In the discussion section, we present the main novelty and principal findings of our work, we discuss the limitations and challenges of our approach and the topic in general. We propose a list of good practice guidelines for using BLE beacons for indoor tracking, based on our experience and insights, and we present a comparison with relevant prior work and a comprehensive comparison across technologies that could be used for indoor location tracking. Finally, we discuss our findings and ways to expand our work in the future, so that such technology applications can pave the development of objective sociability features and eventually support decisions regarding drug efficacy in ASD.

## 2. Methods

### 2.1. Technology and Instructions

**Figure 1:**
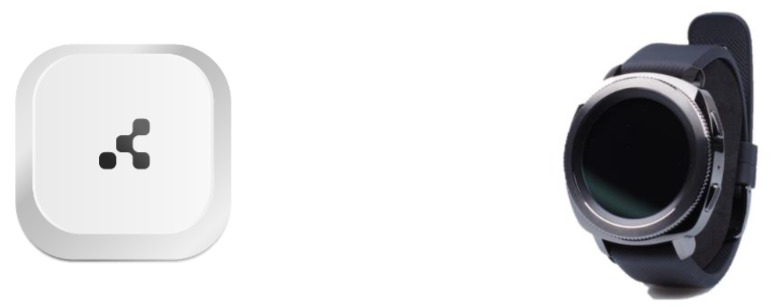
BLE beacons (kontakt.io smart Beacon) deployed in each room and the users’ Samsung Gear Sport smartwatch enable in-home room monitoring.

Multiple beacon transmitting devices (i.e., one BLE beacon for each room), and one receiving device (i.e., one smartwatch) can enable in-home room-tracking of a specific subject. Among the vendor-defined protocols for BLE beacon sensors, we picked iBeacon, which is composed of a Universally Unique Identifier (UUID), a major ID, a minor ID and the transmission power [15]. We used the UUID as an identification label for the beacon and we set the transmission power of all beacons to the maximum value (7) to maximize the chance of receiving signal from the beacons even if there are obstacles in between the receiver and the iBeacon. Figure 1 shows the iBeacon sensor (kontakt.io smart Beacon [16] that can provide over 4 years of battery life) and smartwatch (Samsung Gear Sport [17]) used in the study. Each iBeacon transmits a signal with their UUID at a frequency of 1 Hz. The 1 Hz sampling frequency aims to allow for the maximum amount of received signal, given that collisions, interferences, etc. result in missing values as described in Section 4.2.

Some previous attempts to use iBeacons for indoor tracking followed a calibration-based approach [18,19] to account for complex interference patterns, which is a laborious process that must be repeated every time something changes in the environment (e.g., change of furniture placement). We took a different approach with no signal calibration process for the iBeacons. This made the use of the technology by nonprofessionals (e.g., participants of a clinical trial) easier and still provided valuable insights. We aimed to account for all possible changes in environment in the daily processing of the signal since all changes are always relative and affect all iBeacons in proximity.

No calibration also means that there is no ground truth information about where the sensors were deployed in the homes of the clinical study participants. Therefore, we performed a validation experiment firstly to ensure that the location tracking algorithm could accurately predict the room location, and secondly to identify any potential challenges we need to address.

### 2.2. Studies Where Data Was Collected

#### 2.2.1. Clinical Trial

We provided iBeacons to caregivers and participants in an observational clinical trial (‘A Study to Evaluate Scales for Repetitive and Restricted Behaviors in Children, Adolescents, and Adults with Autism Spectrum Disorder (ASD)’ (NCT03611075) (https://clinicaltrials.gov/ct2/show/NCT03611075 (accessed on 7 July 2021)) with 95 patients diagnosed with ASD and 49 typically developing participants, including children, adolescents and adults. The study has received all necessary Institutional Review Board (IRB) approvals.

The participants and their caregivers received iBeacons for all their rooms in a box. If the smartwatch was switched on and in proximity of all the iBeacons when they were still in the original box as provided by the clinician, then the received beacon signal would be similar (same signal trends) across different iBeacons, because they were all at the same distance from the receiving device (i.e., smartwatch). If the iBeacons were separated (i.e., one in each room), then the signal received from each iBeacon should have different trends. We later evaluate if the iBeacons have been left in their box, or they have been set up one in each room of the household (Section 3.3).

During the first clinical interview, caregivers were asked to classify each room as social, sometimes social or not social, following some basic guidelines. They were instructed to label rooms as not social if they were rarely used for social interactions, as social if they were very frequently used for social interactions, and as sometimes social if they were only occasionally used for social interactions. It is important to note that rooms with the same labels (e.g., parents’ bedroom) might in some households be categorized as social, or in others as not social, as it depends on how each family is using each room and on the communication patterns each family has around the house. Therefore, to account for these differences, we asked the caregivers to subjectively label each room based on the extent to which the householder members use the room for socializing.

We developed a dedicated smartphone application for this study, where the clinician could register the rooms of each participant’s home and assign each room to an iBeacon ID and its respective sociability classification as instructed by the caregiver. Each iBeacon was physically labeled with the room name (e.g., living room). Upon arriving home, the caregiver placed the iBeacons in the corresponding room of the home as the label indicated.

Along with the iBeacons, we also provided a wearable device (i.e., smartwatch) with a preinstalled application that collects the received signal strength from all transmitting iBeacons in proximity. We asked trial participants to wear the smartwatch throughout the duration of the clinical trial during the day (starting from the moment they wake up and for the 6–10 h of the smartwatch’s battery life).

This setup in principle enabled us to determine the rooms in which each participant spent their time through the days of the trial, and possibly infer their sociability over time. Our goal was to infer the amount of time people with ASD spend in social or non-social rooms throughout the day (which could be interpreted as a measure of sociability), as well as any routine behavior patterns observed in their everyday lives.

#### 2.2.2. Internal Study with Healthy Volunteers

We asked 10 healthy volunteers to participate in the technical validation study in 10 different home setup environments and ran 45 test runs. Multiple environments help to account for different setups because the layout of the rooms was different. Some homes might have open spaces combining the kitchen and living room, for example, affecting the signal propagation. Moreover, the material the home is built of may differ. For example, brick walls cause much higher signal attenuation than dry walls. Finally, the perception of each participant on how to place the iBeacons in their home might be different and is a very important variable that needs to be tested.

The technical equipment as well as the instructions provided were identical between the clinical trial and the internal technical validation study. In addition, we developed a smartphone application that would guide participants wearing the smartwatch through each registered room. This application was only used in the internal technical validation study to establish ground truth location data in order to determine the accuracy of the algorithm. Data were collected according to the Declaration of Helsinki.

We retrieved ground truth information during this study in the following way. The participants had a smartphone, a smartwatch and were provided with as many iBeacons as rooms in their household (as in the clinical study). The smartphone and smartwatch were connected via Bluetooth and therefore synchronized with the same time. Both devices were equipped with dedicated applications. The smartwatch application recorded Bluetooth signal, and the smartphone application confirmed that the participants had set up the iBeacons, and instructed them how to perform the location tracking experiment with the relevant ground truth.

**Figure 2:**
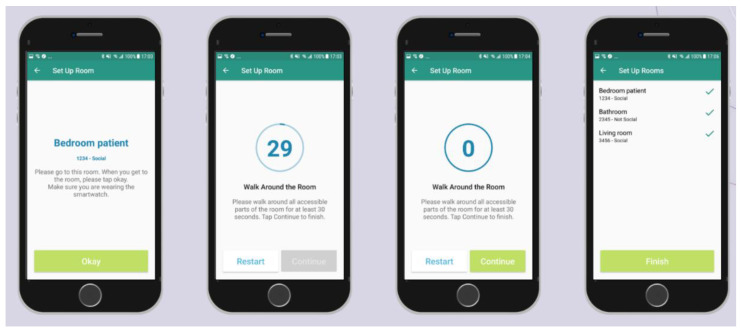
Example screens as shown on the study participant’s smartphone application when performing the experiment of collecting ground truth information during the internal study.

Each iBeacon had to be installed in a specific room as the smartphone application indicated. When the participants performed the experiment, they went to the relevant screen on the smartphone application. The application asked the participants if they were wearing the smartwatch and if they had already set up the iBeacons in the appropriate rooms. If they clicked yes, then the application instructed them to go to a specific room, and click on the screen once they were there (which started the ground truth recording time), as shown in Figure 2. Then a timer of 30 s would start counting down, and once the 30 s were complete, then the participant had to click on the screen again on ‘Continue’ (Figure 2) which would stop the ground truth recording. Then the screen would pop up the message that the person should move to the next room and repeat the same process until all registered rooms in the household have been visited.

**Figure 3:**
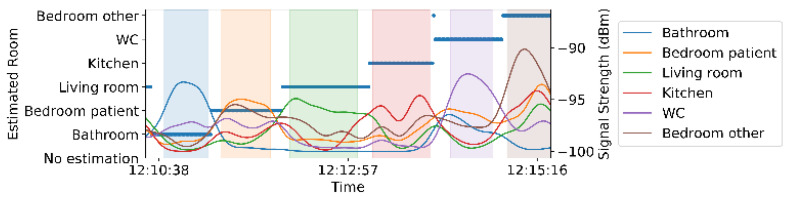
Example visualization of test run number 13 from home setup 8 where the proposed tracking algorithm has 100% accuracy. Ground truth location based on mobile application labeled with shaded background patches; processed beacon data indicated by the colored curves linked to right *y*-axis signal strength in dBm; colors correspond to specific rooms as indicated by the legend; algorithm room location predictions indicated by horizontal blue bars in line with the left *y*-axis.

The Bluetooth signal collected on the smartwatch, and the ground truth location information collected on the smartphone were time aligned during the data analysis phase to assess the accuracy of our proposed location estimation algorithm (using the Bluetooth signal) and compare it against the ground truth location information collected on the smartphone (e.g., Figure 3).

### 2.3. Algorithm

For estimating the closest iBeacon and thereby implicitly the room the person is in, we collected the entire received beacon signal on the smartwatch of each participant whenever they were wearing the smartwatch and performed the following processing of the signal:

- Obtain the raw beacon signal per iBeacon from the smartwatch recording app;
- Resample the beacon signal with frequency of 1 Hz to account for any missing values due to interference/collisions (i.e., when two or more devices attempt to transmit data over a network at the same time);
- Perform linear interpolation per beacon on missing signal gaps below 5 min;
- Fill in any remaining missing data (i.e., for longer intervals than 5 min) with −100 dBm to indicate out of range/missing signal [7];
- Smooth signal per iBeacon using a Gaussian filter with window size of 90 s;
- Rescale signal to the range of [−100, −20] dBm per iBeacon;
- Normalize signal by dividing each iBeacon signal by its mean over the whole day;
- Compute the weight of each iBeacon (range of [0, 1]). The weight of each beacon is its normalized values per time point divided by the sum of the normalized values across all day. If we have in total N iBeacons and this weight is higher than pN = 1/N then the person could actually be in the room with this specific iBeacon;
- Compute and smooth signal derivatives per iBeacon using a Gaussian filter and a window of 90 s.
- Once we have all this information, we use it to estimate the room location for each second in the time range that we have iBeacon data for, as follows:
- Find the iBeacon (bi) with the maximum weight, and the maximum filtered beacon signal strength;
- If the weight of iBeacon bi is greater than threshold pN, and the derivative is not zero, then the estimated room location is set to the room with bi. The non-zero derivative indicates that there is variation in the signal and therefore not missing and replaced by a static value of −100 as described earlier;
- Otherwise, the estimated room location could either be set to unknown (if we are interested only in locations with high confidence), or if we want to avoid having gaps in our location estimation with lower confidence, then the estimated room location is set to the room with the iBeacon with maximum unscaled filtered signal. In our scenario, we are interested in having a continuous signal of room estimations, so we pick the latter approach;
- Once we have a continuous estimation of room locations, we filter out all time points and respective estimated locations while the participant was not wearing the watch. To estimate the spans that the participant was wearing the smartwatch, we were inspired by a previous publication [20]. We filtered out accelerometer data where the standard deviation of Euclidean norm was less than 0.04 m/s^2^ for more than 30 min, as during these spans smartwatches were likely not carried by the subjects. This threshold is higher than the threshold we used for smartphones [20], because the standard deviation of the background accelerometer signal is slightly higher than the smartphone.

### 2.4. Performance Metrics

We used data collected in the internal study to assess the accuracy of the algorithm. We defined as accuracy the percentage of seconds that the room location (defined by the ground truth data) agrees with the iBeacon room estimate (estimated by the algorithm) for each test run. We investigated accuracy as a mean across the 45 test runs, across the average accuracy in the 10 home setups, and across the duration of the ground truth data collected.

In the analysis of the possible sources of error in our system, we explored the number and percentage of occurrences across all identified sources of error, and the median error introduced in our room estimation prediction in seconds.

## 3. Results

### 3.1. Signal Collection

Each iBeacon’s transmission frequency was set to 1 Hz (i.e., a signal per second). As expected, a high amount of missing signal was observed in practice due to the number of iBeacon sensors in close proximity to each other, signal collisions, multipath and attenuation [21]. Moreover, the amount of missing signal and the gap between consecutive received signal measurements also depends on the materials the building is built of and the layout of the space that might encourage more signal collisions [7].

The data from our internal experiment with healthy volunteers and from the clinical trial with both people with ASD and healthy volunteers indicated that the median gap length of signal that needed to be interpolated as the algorithm described in Section 2.3 was 6–8.5 s, depending on the number of beacons per household. Additionally, we estimated that on average; we have received 8.5–20% of the data we would have expected with the theoretical receiving frequency of 1 Hz per iBeacon, validating that the more iBeacon devices in the household, the lower the percentage of received signal.

### 3.2. Performance of the Approach in Healthy Volunteers

The detailed information per household and tests run in our internal study are described in Table 1. The size of rooms across households varies between 15 and 45 m^2^, and the number of rooms in each home setup also reflects the number of iBeacons that were deployed in each scenario. Across 10 home setups and 45 test runs, the average (median) accuracy of the algorithm is 97.2% (99.4%) with standard deviation (STD) of 6.2. Across all home setups, the average (median) accuracy is 94.2% (98.3%) with STD of 8.5. A more detailed breakdown of the impact of wall type and sensor placement on accuracy during our internal study is presented in Table 2. Note that in the case of thin walls, excluding home setup 4 (which might be an outlier) accuracy is still 95.4% (i.e., almost 3% lower compared to concrete walls).

Finally, we see that comparing thin and concrete walls when iBeacons are placed by external walls the accuracy does not vary considerably (98.9% for home setup 1 vs. 99.2% for home setup 6 in Table 1), indicating how placing the sensors in the optimal position might be even more important than the home materials in order to achieve high accuracy with the same system. Note that both home setups 1 and 6 are similar households in terms of furniture placements and number of people living in them.

Figure 3 shows a test run visualization (test run 13 from home setup 8), where the proposed algorithm had 100% accuracy. Ground truth location in Figure 3 is indicated with the shaded background patches; recorded beacon data indicated by the colored curves linked to right *y*-axis signal strength in dBm; colors correspond to specific rooms as indicated by the legend; our algorithm room location predictions indicated by horizontal blue bars in line with the left *y*-axis. We see how the signal strength of each corresponding beacon belonging to each room increases as the participant is entering the room, and decreases as the participant is leaving the room moving to the next one. Signal strength can also fluctuate while in the same room depending on how the participant moves, or if there are other people in the room moving through the line of sight between the sensor and the participant wearing the smartwatch. Note that the ground truth is discontinuous in Figure 3, because when the 30 s of staying in one room are complete (as the application is designed to do and described in Section 2.2.2), then the application instructs the participant to go to the next room, and only once they are in the next room can they press the button that starts recording the current ground truth.

Different setups had different numbers of rooms (on average 4.9 rooms per participant with STD of 1.1), as described in Table 1. All participants had single floor flats, apart from one that had a two-story house (home setup 3). During this experiment, we tested in total 208 rooms, and only one of them was not identified by the proposed location tracking algorithm, suggesting 99.5% sensitivity of detecting a visited room. The average time spent in a room across all home setups and test runs is 35.7 s. The total duration of ground truth information collected across all test runs was 7419 s, and the total duration that rooms were misclassified is 248 s; meaning that the accuracy based on runtime was 96.7%.

Across all ten home setups, only one had mean accuracy below 90% (i.e., home setup 4 with accuracy 69.8%). Table 1 shows that home setup 4 (i.e., test runs 6 and 7) is the only scenario where the iBeacons were placed on the opposite sides of the same very thin walls (i.e., the iBeacons are in very close proximity). With the very low signal attenuation (due to the thin wall), the signal of the two iBeacons is almost the same as if they were placed next to each other. Greater distance between the iBeacons (e.g., placing them in the center of the rooms, or by the external walls as in the other home setups with thin walls as shown in Table 1 and Table 2), could correct for the almost non-existent signal attenuation due to the thin wall material.

We have collected the main reasons observed to lead to room misclassification, and the frequency and effect of these errors on the algorithm is summarized in Table 3. Our results showed that on average we might expect up to approximately 10 s of erroneous room estimation per identified room. However, in the context of our clinical trial application, we were interested in room visits that are longer than a few minutes to describe sociability. Therefore, the algorithm error of 10 s (Table 3) is acceptable.

### 3.3. Feasibility of iBeacon Set Up in a Clinical Trial

We have currently assessed feasibility of beacon setup in 87 of the participants in the clinical trial (‘A Study to Evaluate Scales for Repetitive and Restricted Behaviors in Children, Adolescents, and Adults with Autism Spectrum Disorder (ASD)’ (NCT03611075)). As mentioned in the methodology section, the participants received all their assigned iBeacons in a box during their first clinical visit. If the participant was wearing the smartwatch when all the iBeacons were still in the original box as provided by the clinician, then the received beacon signal would be similar (same signal trends) across all different iBeacons, because they were all at the same distance from the receiving device (i.e., smartwatch). If the iBeacons were separated (i.e., one in each room), then the signal received from each iBeacon should have different trends, due to the signal attenuation and different distances of each iBeacon from the receiving device.

Signal separation indicated that 84 study participants (96.5%) out of the 87, which were provided with iBeacons, set up the iBeacons with one in each room on the first day of wearing the smartwatch, indicating it is feasible for participants to set up the technology without any guidance from a technical person.

**Figure 4:**
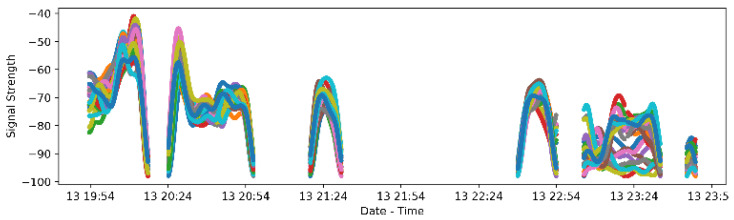
Signal of different iBeacons follow the same trend on the 13th of the month until around 23:00, indicating that they are in the original box provided by the clinician. After 23:00 signals have different trends indicating that iBeacons are set up in different rooms. Note that this is the first day that the participant received the iBeacons, and the devices stayed in the participant’s household for three months (as long as the duration of the clinical study).

Figure 4 presents an example of beacon signal throughout the first day that a participant received the iBeacons. All beacon signals followed the same trend until 23:00, indicating that all beacons were at the same location and distance from the watch (i.e., most likely in the original box of equipment provided by the clinician). After 23:00, we see that different beacon signals had different trends, indicating that they were set up with each one in a different room (different distances from the watch).

The 87 study participants have on average, 6.4 rooms (STD 3.3) with beacon sensors. Out of those rooms, on average 2.6 (40.6%) are social, 1.7 (26.6%) sometimes social and 2.1 (32.8%) not social rooms.

### 3.4. Examples from Individual Participants from the Clinical Trial

**Figure 5:**
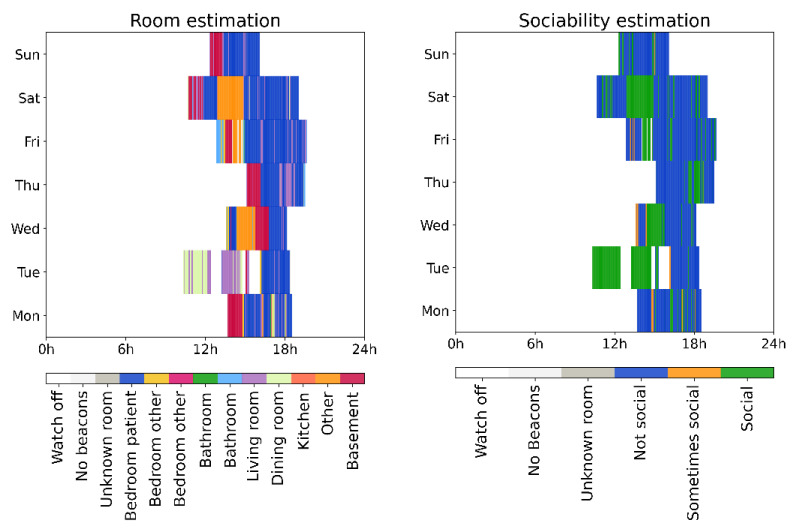
One-week long room (**left**) and sociability (**right**) estimation during the study, from a Monday to Sunday for a participant with ASD. ‘Watch off’ indicates that the participant was not wearing the watch; ‘No beacons’ that the participant has no registered beacon in the proximity wearing the watch, maybe outdoors; ‘Unknown room’ that the participant was at home, but the algorithm could not estimate the room the participant was in with confidence.

**Figure 6:**
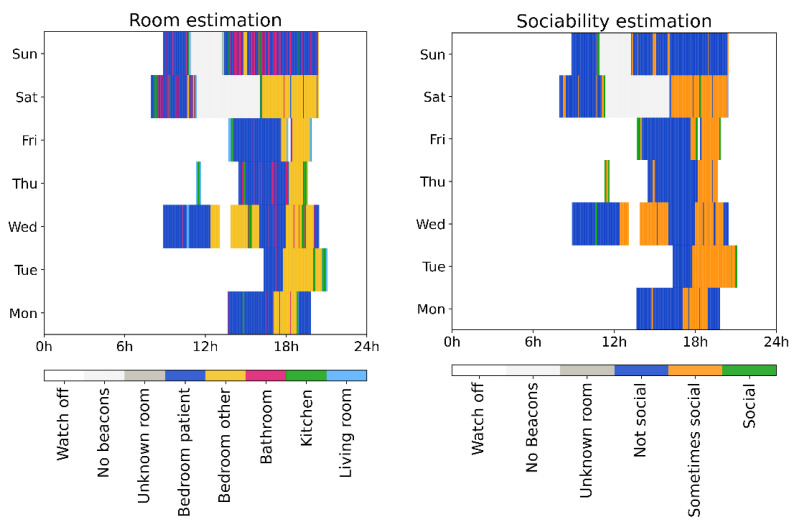
One-week long room (**left**) and sociability (**right**) estimation during the study, from a Monday to Sunday for a different participant with ASD in a different household (hence different rooms and rooms sociability per room compared to Figure 5). ‘Watch off’ indicates that the participant was not wearing the watch; ‘No beacons’ that the participant has no registered beacon in the proximity wearing the watch, maybe outdoors; ‘Unknown room’ that the participant was at home, but the algorithm could not estimate the room the participant was in with confidence.

For the purposes of this work, we used the room and sociability (according to the caregiver’s room sociability labeling) location estimation per participant in the study, as a first step towards the insights we could derive from this kind of data for the everyday socializing patterns of the participants. In the following examples (Figure 5 and Figure 6), we visualized the one-week long location (room and sociability estimation) of two different participants with ASD, with daily location information starting from a Monday during the study to the following Sunday. Each color indicates a different room of the household in the room estimation figures, and the sociability level (i.e., not social, sometimes social and social according to the labeling of the caregivers) of the rooms, in the sociability estimation figures. Moreover, labels ‘Watch off’ indicate that the participant was not wearing the watch; ‘No beacons’ that the participant was wearing the watch, but has not registered beacon in proximity (i.e., maybe outdoors); and ‘Unknown room’ that the participant was at home, but the algorithm could not estimate the room with confidence.

The participant with ASD shown in Figure 5 spends most time in non-social (according to the caregiver) rooms of the house (i.e., the bedroom or the basement). The participant spent mostly short time intervals in social areas such as the living room and another unlabeled rooms, and larger chunks of time in social areas in late afternoons and even longer on Saturday.

A different participant with ASD in a different household, shown in Figure 6, spent most of the time in non-social rooms, such as their bedroom or bathroom. The participant spent short time intervals in social rooms like the living room, and has the pattern of spending time in the evening in another bedroom than their own. We also see that the participant left the house during lunchtime and early afternoon at the weekend wearing the watch, unlike the weekdays.

The complete analysis of the clinical dataset is ongoing and a full report on the clinical study and findings will be published in the near future.

## 4. Discussion

### 4.1. Novelty and Principal Findings

The novelty of this work is twofold. Firstly, to our knowledge this is the first time that BLE beacon data were collected as part of a clinical trial, and where people with no relevant training had to set up such technology without the supervision of a technical person. Secondly, we show that the BLE beacon technology within home monitoring has the potential to provide deep longitudinal insights about the in-home behavior and sociability of study participants in an objective manner, which may overcome shortcomings of subject questionnaire-based measures that are frequently used in ASD and related conditions. When we evaluated the continuous location information collected with BLE technology, our room-tracking algorithm achieves an average accuracy of 97.2% for predicting the correct room across 45 test runs in 10 different household setups. Finally, we identified and reported different possible sources of error for such location tracking algorithms to be taken into consideration for future algorithm and system design.

### 4.2. Limitations

As any real-world technology deployment, our approach comes with certain limitations. The possibility of obtaining reliable ground truth information for each participant’s home setup in a clinical trial setup by a technical person would be impractical and constrained by retaining patient privacy (since an alternative would involve placing cameras in their different rooms).

Throughout all the test runs of our internal study (Section 3.2), we observed three main reasons for room misclassification, and relevant results were summarized in Table 3. Firstly, neighboring rooms. When entering a room, sometimes the previously visited room is still identified for a few seconds due to the short time between room transitions relative to the filtering window. Examples of this kind of error could be from a room that was previously visited as part of our experiment, or a walk-through room during room transition. Secondly, incorrect setup is another important source of error. For example, when two physically neighboring rooms have very thin walls and the iBeacons are placed very close to the opposite sides of the same wall, then there can be room misclassification. Finally, erroneous ground truth data: e.g., user forgets to stop the ground truth recording before moving to the next room (as described in Section 2.2.2).

Another technical challenge was missing data (Section 3.1). A very common reason for missing data in any radio frequency technology is signal collisions. Collisions happen due to interference, which is when more than one iBeacon are transmitting at the same time, trying to acquire the medium/channel simultaneously, resulting in no signal going through to the receiving device [21]. Interference from other devices is unavoidable and cannot be controlled unless a technical person installs the location monitoring system in the study participants’ homes. Therefore, we interpolate missing signal values (due to interference) for a few seconds at a time and during our signal processing.

Missing values also occur when the receiving device (i.e., smartwatch) is out of range of the transmitting device (i.e., iBeacon). This happens if the two devices are too far away, or if there are too many or very dense obstacles in between them. These incidents may be frequent and persistent throughout the study when thick concrete walls block signals from certain rooms, or more transitory due to the positioning of the participant in the room and/or other people blocking the line of sight between beacon and receiver. These factors are out of our control and cannot be predicted. In this case, the missing values of a certain iBeacon are continuous and for longer periods compared to the signal collisions. Therefore, certain beacon sensor locations could misclassify the room location (Table 3). These issues cannot be overcome conclusively while using the proposed technology without introducing additional effort for the participants (calibration etc.), or without trained technical personnel performing beacon sensor setups for each participant.

### 4.3. Sensor Setup Best Practices

The previous section as well as Table 1 and Table 2 show possible scenarios and conditions that may reduce the accuracy of a BLE beacon-based location estimation system. For example, we see that in our limited internal study higher accuracy is achieved with concrete walls and/or by placing the iBeacons as far apart as possible from each other (Table 1 and Table 2). Since it is not always possible to have walls that are not shared with any other room of the household (which provides the highest accuracy no matter the wall type), we suggest that placing the iBeacons in the center of the room is an acceptable choice, as suggested by [22]. To minimize the impact of these and other discussed parameters on room estimation, we put together a list of good practice guidelines that can minimize possible errors due to the home environment, based on what was learnt in the internal study.

Firstly, BLE beacon sensors should be set to have the same transmission power, to make signals of all sensors comparable; meaning that a decrease in signal strength would only indicate that distance to this sensor is increasing or obstacles block the line of sight between the sensors transmitting and receiving. Moreover, using the same transmission power for all beacons helps our system scale in case of a study across many locations. A lower transmission power across all beacons could possibly reduce interference. However, since we cannot know the size of each room and the materials used in each household, we chose to use a high transmission power for all beacons to account for bigger/longer rooms. Secondly, BLE beacon sensors should be placed in an open area in each room that is close to the activity center of the room (to minimize interference). Finally, BLE beacon sensors should ideally have line of sight and face toward the participant (e.g., not placed behind books) and not considerably higher than the receiving device (i.e., smartwatch).

### 4.4. Comparison with Prior Work

Many systems have been developed that aim to estimate the location or activity of people during their everyday lives using sensors. Most common approaches are using GPS [5], Wi-Fi [7,23,24], cameras using computer vision [8], Bluetooth/BLE [9,10,11], ultra wide band (UWB) [25] and accelerometer sensors via dead reckoning [26] (Table 4). GPS is efficient in outdoor location estimation, but not effective indoors. Wi-Fi routers are widely used, but Wi-Fi-based location monitoring relies on knowing the position of the Wi-Fi routers in space, and either requires extensive calibration or training data for fingerprinting [7,24] that cannot adapt to dynamic environments, and extensive manual labor is required or uses time-of-arrival (ToA)/time difference of arrival (TDoA) algorithms [23], where accuracy is limited by the bandwidth of radio signal and clock synchronization. UWB can provide sub-millimeter accuracy when there is line of sight, but the sensors are very expensive for large-scale deployment, and an application on the smartphone of the user needs to have the building information and exact UWB sensor location to perform location estimation. Accelerometers and dead reckoning are easy to deploy as all smartphones/smartwatches have accelerometers. However, the sensors are noisy from their nature, and error accumulation significantly impacts location estimation even if anchor points from more stable technologies are used [26].

Conversely, our proposed approach using BLE sensors requires no prior environment information other than what room each sensor is placed in; is easy to deploy as all smartphones/smartwatches can receive BLE signals and no calibration or training data is needed, making our system able to cope with dynamic environments. Moreover, as Table 4 indicates, BLE technology needs no further information (e.g., floorplan or the precise sensor location) for room level location estimation. Finally, since the signal of the BLE sensors indicates the room, the location estimation happens on the wearable or smartphone device of the users, preserving their privacy from a centralized system (which would require clock synchronization across all devices, adding extra complexity).

Kabelac et al. presented the most relevant prior work in the context of clinical trials and healthcare, showing the potential of a specialized hardware device that can perform passive monitoring in one room at a time [27]. The device used in this study was transmitting ultra-low-power radio signals that reflect off individuals within a range, and could very accurately track one person in the room and their activities. However, this approach required information about each room and even the location of furniture. Moreover, it would be particularly challenging to monitor one study participant when multiple people are in the room, and even harder to monitor the participants continuously as they move from one room to another, both of which comprise key aspects of sociability within the home. Our approach, in comparison to [27], was not limited to one room. Instead, it could always follow the study participants across different rooms, since they are the ones wearing the smartwatch. Moreover, our proposed approach could identify the study participant no matter how many other people are present in the same room. Finally, the hardware needed to be deployed in each room in our approach was a low-cost consumer BLE beacon sensor instead of a specialized device that is not available to the public yet. Overall, our approach is practical, reliable in monitoring the study participant across the whole house and easily deployable.

Another healthcare application of indoor location monitoring was proposed by Urwiler et al., for older adults with and without dementia, with the goal to recognize activities of daily living [28]. The technology used in this work was passive infrared motion sensors, which has no battery constraints or signal interference issues to impact accuracy. However, their system needed to be set up in the home of each study participant by a technical person, meaning that, compared to our system, deployment is more complex.

Surian et al., used the number of signals from BLE beacons to identify the user’s room location, assuming that the signal propagation remains the same in different environments, which is unrealistic [29]. The authors only reported the performance of their system in two test runs with only one participant and average accuracy of approximately 81.5%. Our algorithm outperforms their accuracy by 15%, without any assumptions and with multiple test runs and participants.

Kyritsis et al., leveraged BLE sensors to efficiently heat a house, room by room, based on the habitants’ habits and preferences, by placing a BLE sensor in the center of the ceiling of each room [22]. The proposed algorithm estimates received signal strength indicator (RSSI) thresholds that would indicate if the participant is in the room with a certain BLE sensor, by using the room dimensions. Our approach is more agile, as it can detect the room location with high accuracy without requiring any a priori knowledge of the environment.

Finally, there is a considerable amount of prior work focusing on using calibration or training data (e.g., [18,19]). However, such approaches fail to adapt in dynamic environments, requiring new rounds of strenuous calibration that non-technical people cannot easily perform, unlike our lightweight approach.

### 4.5. Learnings and Future Work

The internal study provided many insights and improvements for our methodology. Regarding the iBeacon placement, no concerns were reported by the internal study participants. The ones that could not place the iBeacon sensors in the center of the room placed them in a more convenient location (Table 1 and Table 2). This was helpful for our analysis because we could evaluate our algorithm in real-world non-optimal conditions and validate that the overall accuracy remains high. Moreover, we could identify possible issues that arise due to beacon placement (Table 2 and Table 3) and eventually propose optimal placement guidelines (Section 4.3). We are also collecting relevant feedback from the clinical study participants to include in a future publication focusing only the clinical trial, together with more challenges (technical or not) that came up in the course of the study.

Feedback from users in the clinical study was highly encouraging. Most participants found the system easy to set up and non-invasive during daily life. The most common complaints related to a small number of participants finding the wearable uncomfortable. This is a first attempt towards solving the issue of indoor location estimation in the context of a clinical trial, and we will be looking into more unobtrusive methods in the future.

In this work, we have focused on the beacon data, but we also make use of accelerometer data (collected together with beacon data, using the same wearable) to assess if the person is wearing the watch, as described in Section 2.3. Additional sensor data were captured from the smartwatch sensors relating to movements and physiology during the clinical study. These data are included in the ongoing analysis of the clinical study data.

Our proposed approach can quantify the time the study participants spend in rooms labeled as social, sometimes social or not social by their caregivers, but cannot know if there were other people in the same room. As a first step to address this issue, we have introduced and are currently exploring the feasibility of using “people beacons” (i.e., small beacon sensors that family members carry on them at all times during the study). We plan to explore this option further in the future.

Finally, although our focus has been to develop and test this technology within the homes of people with ASD, this technology and algorithm could be deployed in any scenario where at home monitoring could be of interest. Such applications might include within the homes of elderly residents or people with depressive disorders.

## 5. Conclusions

In this work, we used BLE beacon technology for indoor passive location tracking in the context of a clinical trial. We developed and described a room-tracking algorithm that identified the correct room for the study participant with 97.2% accuracy in our internal performance evaluation. Moreover, we showed that it is feasible to deploy this technology in a clinical trial without any technical assistance or calibration/training data collection. Participants were adherent with early set up of the BLE beacon sensors (96% of them performing it on the first day). We showed that beacon room-tracking in clinical trials could provide insights into the patients’ everyday sociability patterns. Finally, we proposed a set of good practice guidelines for using BLE beacon technology in the future.

As a next step, we plan to develop BLE beacon-based features, to identify meaningful associations with clinical markers related to sociability in the context of ASD.

## Figures and Tables

**Table 1:** Home setups in our internal study with healthy volunteers, number of rooms (matching the number of iBeacons deployed in each home setup) and room estimation accuracy results for different home setups. Note than thin wall type is meant to include both wooden and plaster walls.

Home Setup	Number of Rooms	Test Run	Test Run Accuracy (%)	Mean Setup Accuracy (%)	Wall Type	iBeacon Location in Room
1	3	1–2	97.9, 100	98.9	Concrete	By external walls
2	5	3–4	97.7, 99	98.4	Thin	Center
3	7	5	92.8	92.8	Thin	Center
4	6	6–7	69.4, 70.3	69.8	Thin	By shared walls
5	5	8–9	93.9, 96.4	95.2	Concrete	Center
6	4	10	99.2	99.2	Thin	By external walls
7	4	11	91.4	91.4	Thin	Center
8	6	12–17	96.2, 100, 100, 100, 97.3, 100	98.9	Concrete	Center
9	4	18–38	97.7, 100, 100, 100, 97.7, 100, 100, 100, 100, 99.4, 100, 98.5, 99.4, 100, 97.7, 97.9, 100, 98, 100, 100, 100	99.3	Concrete	Center
10	5	39–45	95.2, 97.5, 99.4, 100, 98.3, 100, 97.6	98.3	Concrete	Center

**Table 2:** Impact of wall type and sensor placement on accuracy during our internal study. Note that the number of home setups is some cases (e.g., iBeacon placement by external/shared walls) is very low and is only an indication of possible performance.

Wall Type	iBeacon Location in Room	Number of Home Setups	Mean Accuracy (%)
Concrete	-	5	98.1
Thin	-	5	90.3
-	Center	7	96.3
-	By external walls	2	99.1
-	By shared walls	1	69.8

**Table 3:** Summary of sources of error during the internal study.

Reason for Room Misclassification	Number of Occurrences	Percentage of Occurrences out of All Sources of Error	Median Error per Room Estimation
Previously visited as part of the test run	13	33.3%	2 s
Next visited as part of the test run	11	28.2%	2 s
Walk by/through room during room transition	6	15.4%	1 s
Neighboring rooms have very thin walls & iBeacons not in the center of each room	4	10.3%	11 s
Error with start/end ground truth recorder	5	12.8%	5 s

**Table 4:** Summary of technologies that could be used for indoor location estimation. GPS is not included as it is only effective outdoors, and IMU is not included as it only provides relative movement information if not combined with one of the following technologies. Note that ‘centralized’ refers to if the location estimation happens on a ‘server’ instead of on a mobile device itself. ‘Maybe’ indicates that in different applications of the technology it could satisfy the specific criterion or not.

Technology	Calibration Needed	Sensor Exact Location in Room Needed	Centralized (Synchronization Needed)	Privacy Preserving	Off-the-Shelf Hardware
Wi-Fi	Yes	Maybe	Yes	Maybe	Maybe
Bluetooth/BLE	No	No	No	Yes	Yes
Cameras	Yes	Yes	Yes	No	Yes
UWB	No	Yes	Yes	Maybe	No

## Data Availability

Qualified researchers may request access to individual patient-level data through the clinical study data request platform (https://vivli.org/ (accessed on 7 July 2021)). Further details on Roche’s criteria for eligible studies are available here (https://vivli.org/members/ourmembers/ (accessed on 7 July 2021)). For further details on Roche’s Global Policy on the Sharing of Clinical Information and how to request access to related clinical study documents, see here (https://www.roche.com/research_and_development/who_we_are_how_we_work/clinical_trials/our_commitment_to_data_sharing.htm (accessed on 7 July 2021)).
